# PTB Domain-Directed Substrate Targeting in a Tyrosine Kinase from the Unicellular Choanoflagellate *Monosiga brevicollis*

**DOI:** 10.1371/journal.pone.0019296

**Published:** 2011-04-26

**Authors:** Victoria Prieto-Echagüe, Perry M. Chan, Barbara P. Craddock, Edward Manser, W. Todd Miller

**Affiliations:** 1 Department of Physiology and Biophysics, School of Medicine, Stony Brook University, Stony Brook, New York, United States of America; 2 sGSK group, Neuroscience Research Partnership/A*Star, Singapore, Singapore; University of Connecticut, United States of America

## Abstract

Choanoflagellates are considered to be the closest living unicellular relatives of metazoans. The genome of the choanoflagellate *Monosiga brevicollis* contains a surprisingly high number and diversity of tyrosine kinases, tyrosine phosphatases, and phosphotyrosine-binding domains. Many of the tyrosine kinases possess combinations of domains that have not been observed in any multicellular organism. The role of these protein interaction domains in *M. brevicollis* kinase signaling is not clear. Here, we have carried out a biochemical characterization of *Monosiga* HMTK1, a protein containing a putative PTB domain linked to a tyrosine kinase catalytic domain. We cloned, expressed, and purified HMTK1, and we demonstrated that it possesses tyrosine kinase activity. We used immobilized peptide arrays to define a preferred ligand for the third PTB domain of HMTK1. Peptide sequences containing this ligand sequence are phosphorylated efficiently by recombinant HMTK1, suggesting that the PTB domain of HMTK1 has a role in substrate recognition analogous to the SH2 and SH3 domains of mammalian Src family kinases. We suggest that the substrate recruitment function of the noncatalytic domains of tyrosine kinases arose before their roles in autoinhibition.

## Introduction

The machinery necessary for phosphotyrosine-based signaling in metazoans includes “writer” domains (tyrosine kinases), “readers” (SH2 and PTB domains), and “erasers” (tyrosine phosphatases) [1], [2], [3]. Genome analyses suggest that “eraser” domains emerged earliest in evolution; examples of tyrosine phosphatases can be found, for example, in the yeast *S. cerevisiae*. Metazoans have large numbers of SH2, PTB, tyrosine kinase, and tyrosine phosphatase domains, suggesting a concerted expansion of the apparatus needed for phosphotyrosine signaling. Surprisingly, the genome of the unicellular choanoflagellate *Monosiga brevicollis* contains numbers of each of these domains that are comparable to complex multicellular organisms [3], [4], [5]. Because choanoflagellates are considered to be the closest living unicellular relatives of metazoans [5], [6], [7], the *Monosiga brevicollis* genome affords an important glimpse into the early evolution of tyrosine kinases and phosphatases.

In addition to their catalytic domains, metazoan nonreceptor tyrosine kinases (NRTKs) possess noncatalytic domains that are important in kinase function [8], [9], [10]. For example, the SH3 and SH2 domains of Src-family tyrosine kinases have two important functions: they participate in intramolecular interactions that regulate the kinase domain, and they target the enzyme to cellular substrates by specific protein-ligand interactions [11], [12]. Many of the NRTKs in *Monosiga brevicollis* display combinations of domains that are not observed in multicellular animals [2], [3]. Among the unique domain combinations are kinases containing C2, FYVE, and PTB domains. These observations underscore the importance of domain shuffling in the emergence of tyrosine kinase signaling. Studies on two Src-related kinases from *Monosiga brevicollis* (MbSrc1 and MbSrc4) have suggested that the substrate targeting function of the SH3 and SH2 domains evolved earlier than the ability of the domains to engage in autoinhibitory interactions [13], [14].

The *Monosiga brevicollis* genome contains fifteen HMTK kinases [3]. (The name HMTK is an acronym for HM motif-containing tyrosine kinase, so named because the conserved His-Arg-Asp sequence within the catalytic loop is replaced by a His-Met sequence in this family). Ten of the fifteen HMTK kinases contain one or more PTB domains, which in multicellular organisms often bind to phosphotyrosine-containing proteins [15]. The HMTK kinases are of particular interest, because the PTB domains may play analogous functions to the SH2 domains found in many families of nonreceptor tyrosine kinases; for example, the PTB domains may be involved in targeting the HMTK kinase domain to cellular proteins for phosphorylation [3]. Thus, HMTK kinases may represent an example of convergent evolution. In this paper, we have cloned and characterized the PTB-containing HMTK1 kinase. We report that the enzyme is active, and that the PTB domain binds to peptides that contain phosphotyrosine. HMTK1 preferentially phosphorylates a substrate containing a PTB ligand, suggesting that this system represents an early example of substrate targeting.

## Materials and Methods

### Antibodies and other reagents

Anti-phosphotyrosine antibody (clone 4G10) was from Millipore, anti-Flag M2 and anti-tubulin clone GTU-88 were from Sigma, mouse monoclonal anti-His_6_ was from Covance, and anti-GST was from Molecular Probes. Horseradish peroxidase-linked sheep anti-mouse IgG antibodies were from GE Healthcare. Leupeptin, aprotinin, PMSF, sodium vanadate, sodium fluoride, pyruvate kinase/lactate dehydrogenase enzymes, reduced NADH, ethanolamine, and EZview red anti-Flag M2 affinity gel were from Sigma. Affi-gel 15 agarose was purchased from BioRad.

### cDNA cloning and mutagenesis

The predicted sequence of HMTK1 (784 amino acids) was obtained from the Joint Genome Institute gene model (NCBI accession number: XM_001749555). The form of HMTK1 used in this study was amplified by PCR from a *M. brevicollis* cDNA library [16] using the 5′ primer ATCATGGGCGTCTTTGAAGCCACC and the 3′ primer GCTCTAGATCAATTCCTGTGCC-ATGTTGGCAAAGGATGGGCG. The 5′ primer binds at the start of the third PTB domain of HMTK1 (residue 341); we were not successful in amplifying a cDNA using primers at the 5′ end of the gene model, or at the beginning of the first or second PTB domains. The 3′ primer extends to Leu 761 of the predicted sequence. PCR reactions using a 3′ primer based on the entire 784 amino acids were unsuccessful. Thus, the 23 C-terminal amino acids from the gene model are missing. These residues are not predicted to be in a conserved domain.

For expression in insect cells, HMTK1 DNA (encoding residues 341–761) was cloned into the *Eco*RI site of pFastbac-Hta (Invitrogen). FLAG-tagged HMTK1 was expressed in mammalian cells by subcloning into the BglII and BamHI sites of p3XFLAG-CMV (Sigma). The ΔPTB version of this construct was prepared by PCR amplification and subsequent recloning into the BamHI and *Eco*RI sites of p3XFLAG-CMV. Site-directed mutagenesis of FLAG-HMTK1 was carried out using the QuikChange kit (Stratagene). To express the isolated third PTB domain (residues 341–474) in bacterial cells, this PCR fragment was cloned into the *Eco*RI site of plasmid pGEX-4T-1 (GE Healthcare). All constructs were confirmed by DNA sequencing.

### Protein expression and purification

HMTK1 was expressed in *Spodoptera frugiperda* (Sf9) insect cells using the Bac-to-Bac system (Invitrogen). Sf9 cells (800 ml) were infected with HMTK1 baculovirus and harvested after three days. Cells were lysed in a French pressure cell, and His-tagged HMTK1 was purified using nickel-nitrilotriacetic acid resin (Qiagen), as described previously [13]. Peak fractions were pooled and concentrated in an Amicon Ultrafiltration device (molecular weight cutoff: 30,000 daltons). The purified protein was stored in 40% glycerol at −20°C. The isolated PTB domain of HMTK1 was produced as a fusion with glutathione S-transferase (GST). *E. coli* cells (1 liter) expressing the GST fusion protein were lysed in a French pressure cell, and the GST-PTB protein was purified by chromatography on a glutathione-agarose column (Molecular Probes).

### Tyrosine kinase assays

Kinase assays were performed by two methods. Initial activity measurements were performed by the phosphocellulose paper binding assay [17]. Reaction mixtures contained 20 mM Tris-HCl (pH 7.4), 10 mM MgCl_2_, 0.2 mM ATP, [γ-^32^P]-ATP (30–50 cpm/pmol), and varying amounts of HMTK1 and peptide substrates. All peptide substrates were purified by high pressure liquid chromatography and characterized by mass spectrometry prior to use. The following substrates were used: c-Src peptide, AEEEIYGEFEAKKKG; MbSTAT peptide, KKKASGYVMADIA; RTKB2 peptide 1, SEEVYGAVVDKKK; RTKB2 peptide 2, AEEVYEAIADKKK; SH2 binding substrates (varying linker lengths), RRLEDAIYAAGGGGGEPPQpYEEIG, RRLEDAIYAAGEPPQpYEEIG, and RRLEDAIYAPQpYEEIG; SH2 control substrate, RRLEDAIYAAGGGGGEPPQFEEIG. Initial rate kinetic measurements on the PTB ligand-containing substrate (KKAEEEIYGEFEANFTNPVpYATLG) and on the Phe-containing PTB control (KKAEEEIYGEFEANFTNPVFATLG) were carried out using a continuous spectrophotometric assay [18]. Reactions were performed at 30°C in a final volume of 50 µl. The reactions contained 100 mM Tris pH 7.4, 10 mM MgCl_2_, 2 mM ATP, 1.5 mM phosphoenolpyruvate, 90 units/ml of pyruvate kinase, 109 units/ml of lactate dehydrogenase, and 1.2 mg/ml of NADH. For determination of K_m_ values, the enzyme concentrations were 0.3–0.5 µM and the peptide concentrations ranged from 0–700 µM. Kinetic constants were determined by nonlinear fitting to the [substrate] vs. velocity curves using GraphPad Prism 4.

### Peptide binding reactions

The PTB ligand-containing peptide, Phe-containing PTB control, and pYEEI peptide (EPQpYEEIG) were linked to Affi-Gel 15 resin (Bio-Rad) following the manufacturer's protocols. Purified HMTK1 or the isolated PTB domain were incubated for 1 hour at 4°C with the immobilized peptides. After binding, the beads were washed 4 times with 1 ml of binding buffer (50 mM Tris-HCl pH 7.5, 5 mM EDTA, 100 mM NaCl, 0.1% Triton X-100, 1 mM DTT, 0.5 mM Na_3_VO_4_). Bound proteins were eluted with SDS-PAGE loading buffer, separated by 10% SDS-PAGE, and visualized either by Coomassie blue staining or by Western blotting.

### Biotinylation of HMTK PTB domain

The PTB domain of HMTK1 was cloned as an EcoR1 fragment into a modified pGEX (GE Healthcare Life Sciences) bicistronic vector pGEX-4T-BiotinN to express the gene of interest and the biotin ligase (BirA) in the same cell ([19]; Chan et al., in press). This results in a GST-PTB fusion protein biotinylated at the 10-residue acceptor sequence IFEAQKWMEWRggs (biotin target residue underlined; spacer sequence in small case) that is part of the linker region between the GST and PTB domains. The resulting pGEX-BiotinN-PTB construct was transformed into *E. coli* BL21(DE3) cells for expression. GST-biotin-PTB protein was purified using glutathione-agarose and desalted using a PD-10 (GE Healthcare) gel filtration column.

### Peptide array experiment

N-terminally acetylated 11-residue peptides were synthesized using standard chemistry in situ (PepSpots) on cellulose based matrix (Jerini Biotools). To ensure protein accessibility, each peptide contained a 4-residue spacer consisting of a glycine and 3 β-alanines. All peptides were immobilized via their C-termini. To assess synthesis reproducibility, six different phosphopeptide sequences predicted for 14-3-3 binding were made in duplicate in different rows on the array, and displayed less than 10% standard error for 14-3-3 binding levels between identical sequences. Prior to usage, peptide array membranes were washed in binding buffer (20 mM Hepes pH 7.3, 137 mM NaCl, 5 mM KCl, 0.05% Tween-20) and blocked with 5% filtered bovine serum albumin in the same buffer. Recombinant biotinylated PTB protein was diluted to 10 µg/ml in binding buffer containing 5% BSA, and incubated with the array membrane for 30 min at room temperature. After washing (2×10 min), streptavidin-HRP (1∶20,000, GE) was added for 15 minutes at RT. After washing in binding buffer (3×10 min), bound PTB was detected using enhanced chemiluminescence and standard X-ray film.

### Cell transfections, immunoprecipitation and Western blotting

SYF cells were cultured in Dulbecco's modified Eagle's medium (Mediatech, Inc.) supplemented with 10% fetal bovine serum (Sigma) and 1000 UI/ml penicillin, 1000 UI/ml streptomycin and 25 ng/ml amphotericin B (Mediatech, Inc.). SYF cells (2.25×10^6^) were plated in 15-cm diameter dishes and transfected after 24 hours using 5–15 µg of DNA and 2 µl TransIT per µg DNA. After 48 hours, the cells were harvested, washed twice with PBS and lysed using radioimmune precipitation assay (RIPA) buffer (50 mM Tris-HCl pH 7.4, 150 mM NaCl, 5 mM EDTA, 1% sodium deoxycholate, 1% Nonidet P-40) supplemented with the protease inhibitors leupeptin (10 µg/ml), aprotinin (10 µg/ml), PMSF (200 µM) and the phosphatase inhibitors Na_3_VO_4_ (0.2 mM) and NaF (10 mM). Lysates (50 µg) were separated by 10% SDS-PAGE, transferred to PVDF membranes and probed with the appropriate antibodies. For immunoprecipitation, cell lysates (100 µg) were incubated overnight at 4°C with 40 µl of EZview red anti-Flag M2 affinity gel pre-equilibrated with RIPA buffer in a total volume of 1 ml. The immunocomplexes were washed 4 times, eluted from the beads by adding Laemmli buffer, separated by SDS-PAGE, transferred to PVDF membranes and probed with the appropriate antibodies.

## Results

**Figure 1:**
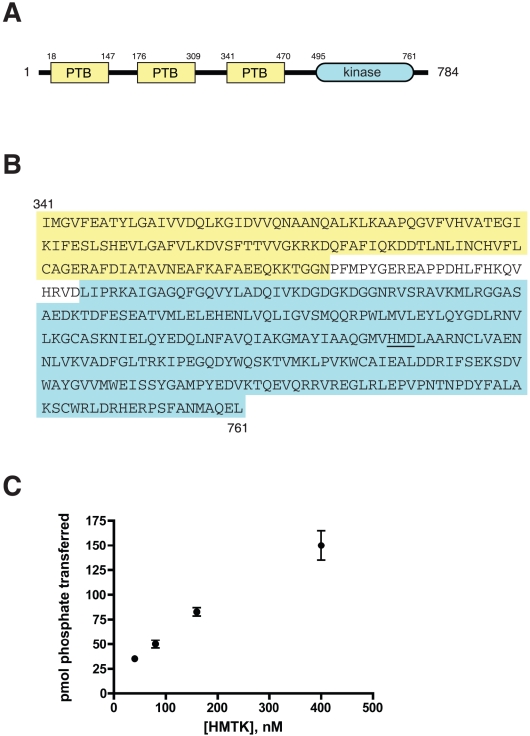
*Monosiga brevicollis* HMTK1 kinase. (A) Domain arrangement of HMTK1. (B) Amino acid sequence of the PTB-kinase HMTK1 construct used in this study. The HMD motif in the catalytic loop is underlined. (C) Tyrosine kinase activity of HMTK1. Purified HMTK1 (at the indicated concentrations) was assayed with the c-Src peptide (500 µM). Reactions proceeded for 6 minutes at 30°C, and enzyme activity was measured using the phosphocellulose paper assay.

The predicted amino acid sequence of *M. brevicollis* HMTK1 contains three PTB domains and a C-terminal tyrosine kinase catalytic domain (Fig. 1A). We amplified a cDNA encoding residues Ile341-Leu761 by PCR from an *M. brevicollis* cDNA library. This construct contains the third PTB domain plus the kinase domain, and lacks the predicted C-terminal 23 amino acids (Fig. 1B). We were unable to amplify cDNAs encoding the first or second PTB domains, or the extreme C-terminus, suggesting that these longer forms are not expressed, at least under the conditions used to generate the cDNA library. There is a predicted intron/exon boundary in the HMTK1 gene two codons upstream of the third PTB, raising the possibility that this single-PTB form of HMTK1 is expressed.

The third PTB domain shows highest homology to the Gulp and Numb PTB domains (e.g., 31% amino acid identity with the mouse Gulp-2 protein). The kinase domain is most closely related to the fibroblast growth factor receptor-1 tyrosine kinase (39% amino acid identity with the human FGFR1; Figure S1). HMTK1 possesses most of the catalytically important sequence elements that are conserved across the protein kinase superfamily. HMTK1 has the kinase-conserved DFG motif (at Asp647) that is involved in ATP binding. The predicted activation loop of HMTK1 contains a single tyrosine (Tyr660, in the sequence EGDQYWQSK), with the N-terminal residues to the tyrosine typical for autophosphorylated acidophilic kinases. However, the conserved HRD motif in the catalytic loop is replaced with HMD (Fig. 1B). The arginine within the HRD motif typically interacts with phosphate in protein kinases that are regulated by activation loop phosphorylation [20], [21]; however, it is possible that the HMTK1 His residue could play an analogous role.

**Figure 2:**
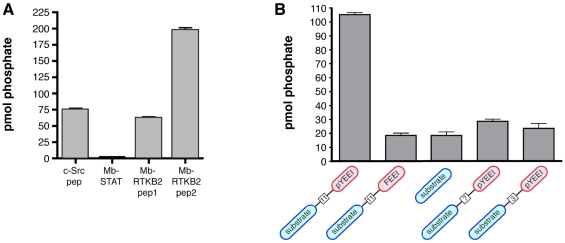
Additional HMTK1 peptide phosphorylation assays. Reactions proceeded for 6 minutes at 30°C, and enzyme activity was measured using the phosphocellulose paper assay. (A) Phosphorylation of a c-Src peptide and Monosiga-derived peptides (750 µM) by purified HMTK1 (400 nM). The sequences are: c-Src peptide, AEEEIYGEFEAKKKG; MbSTAT peptide, KKKASGYVMADIA; RTKB2 peptide 1, SEEVYGAVVDKKK; RTKB2 peptide 2, AEEVYEAIADKKK. (B) Phosphorylation of SH2-binding substrates containing various spacer lengths between the pY residue and the phosphorylatable tyrosine. The peptides are shown schematically, with the phosphorylation site in blue, the SH2-binding ligand (pYEEI) in red, and the number of intervening amino acids in a box. The sequences of the peptides are: RRLEDAIYAAGGGGGEPPQpYEEIG (spacer = 11), RRLEDAIYAAGEPPQpYEEIG (spacer = 7), and RRLEDAIYAPQpYEEIG (spacer = 3); SH2 control substrate, RRLEDAIYAAGGGGGEPPQFEEIG (spacer = 11); substrate peptide, RRLEDIAYAAG. Reactions contained 70 µM peptides. Error bars show standard deviation.

To test whether HMTK1 is enzymatically active, we cloned the HMTK1 DNA into a baculovirus expression vector and expressed the enzyme in *Spodoptera frugiperda* (Sf9) cells. We purified the His-tagged PTB-kinase construct using nickel-nitrilotriacetic acid resin. For our initial enzymatic characterization, we measured phosphorylation of an acidophilic Src peptide (AEEEIYGEFEAKKKKG) [22] using ^32^P-labeled ATP (Fig. 1C). HMTK1 phosphorylated this peptide efficiently, and the activity showed the expected dependence on enzyme concentration. HMTK1 displayed no activity toward peptide substrates for Ser/Thr-protein kinases (data not shown), confirming that it is a tyrosine-specific protein kinase. Next, we compared phosphorylation of this peptide with peptides derived from putative *M. brevicollis* kinase substrates. Two of the peptides (RTKB2 peptides 1 and 2) correspond to sequences from the intracellular domain of a *M. brevicollis* receptor tyrosine kinase, and the third (MbSTAT) is from a putative *M. brevicollis* STAT [3]. HMTK1 showed highest activity towards peptide RTKB2 peptide 2, roughly equivalent activity towards RTKB2 peptide 1 and the c-Src substrate peptide, but no significant activity towards MbSTAT (Fig. 2A). The two *Monosiga* kinases previously characterized (MbSrc1 and MbSrc4) had substantially higher activities towards RTKB2-1 and RTKB2-2 compared with the c-Src peptide [13], [14], suggesting that the kinase domains possess a measure of intrinsic substrate specificity.

By anti-phosphotyrosine Western blotting, the preparation of HMTK1 shows evidence of phosphorylation (Fig. S2). (This could be due to HMTK1 autophosphorylation, or to phosphorylation by endogenous Sf9 cell kinases). Treatment of purified HMTK1 with Yersinia tyrosine phosphatase led to a decrease in phosphorylation. Incubation of HMTK1 with ATP and MgCl_2_ under conditions that typically promote autophosphorylation of tyrosine kinases (e.g., [23]) did not increase the pTyr signal, suggesting that the autophosphorylation activity of HMTK1 is relatively weak.

We measured HMTK1 activity toward a series of peptide substrates which incorporated the Src SH2 ligand pYEEI. For Src-family kinases, the presence of the pYEEI sequence leads to a ≈10-fold reduction in peptide K_m_, due to SH2 domain-mediated targeting [24]. A substrate peptide possessing the pYEEI sequence was phosphorylated 5-fold more strongly than a control sequence lacking phosphotyrosine or a shortened peptide containing only the substrate motif (Fig. 2B). These results suggest that the PTB domain of HMTK might recognize pYEEI. The SH2-binding substrate used in Fig. 2B had a spacer of 11 residues between the pYEEI sequence and the phosphorylatable tyrosine. We tested HMTK1 with peptides containing shorter linker lengths, but we did not observe preferential phosphorylation of these peptides relative to the control (Fig. 2B); this result is similar to results with Src-family kinases [24].

**Figure 3:**
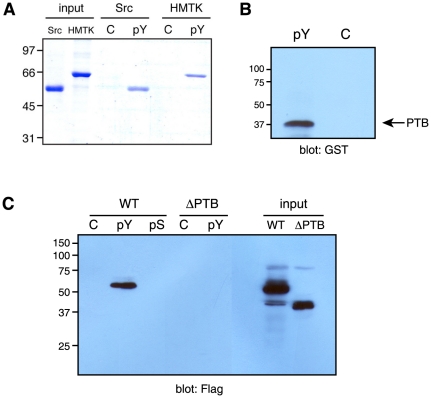
Binding reactions with pYEEI-containing peptide. (A). Purified HMTK1 or c-Src (20 µg) were mixed with 30 µl of immobilized pYEEI peptide (pY) or control resin (C) in total volumes of 175 µl for 1 hour at 4°C. After binding, the resins were washed 4 times with 200 µl of binding buffer. Bound proteins were eluted with SDS-PAGE sample buffer and analyzed by gel electrophoresis with Coomassie blue staining. (B). The isolated HMTK1 PTB domain (8 µg) was incubated with 50 µl of immobilized pYEEI peptide (pY) or control resin (C) in total volumes of 400 µl for 1 hour at 4°C. After washing, bound proteins were eluted with SDS-PAGE sample buffer and analyzed by Western blotting with anti-GST antibody. (C). Lysates from SYF cells expressing FLAG-tagged wild-type or ΔPTB forms of HMTK1 were incubated with immobilized pYEEI peptide (pY), phospho-Kemptide (pS), or control resin. After washing, bound proteins were analyzed by anti-FLAG Western blotting. The input lanes show lysates (corresponding to 20% of the amounts used for pulldowns) loaded directly on the gel.

We tested whether HMTK1 could interact with phosphotyrosine in a direct binding assay with immobilized pYEEI (Fig. 3A). Purified HMTK1 (or as a positive control, purified Src) was mixed with pYEEI peptide that had been attached to Affi-Gel 15 resin. Both HMTK1 and Src bound to pYEEI in this experiment, but did not interact with the Affi-Gel control resin. We confirmed that the pYEEI-binding activity was localized to the PTB domain by using the isolated PTB domain in a pulldown assay (Fig. 3B). We produced a version of HMTK1 lacking the PTB domain (ΔPTB). FLAG-tagged forms of wild-type HMTK1 and ΔPTB were expressed in triple Src/Yes/Fyn-knockout (SYF) cells [25], and lysates were used in pulldown experiments. Wild-type HMTK1 bound to immobilized pYEEI, while ΔPTB did not (Fig. 3C). HMTK1 failed to bind to an immobilized phosphoserine-containing peptide (phospho-Kemptide, LRRApSLG), suggesting that the negatively charged phosphate of pYEEI was not the sole binding determinant.

In the next series of experiments, we tested whether HMTK1 possesses tyrosine kinase activity towards mammalian protein substrates. We expressed FLAG-tagged HMTK1 in SYF cells to reduce background phosphorylation. For comparison, we also expressed ΔPTB, as well as two point mutants containing amino acid substitutions that could potentially disrupt PTB-phosphotyrosine interactions (Figure S3). Anti-phosphotyrosine Western blotting of SYF cell lysates showed no significant activity for HMTK1 (wild-type or mutants) over the background levels seen in untransfected SYF cells (Figure S4A). Treatment of SYF cells with sodium orthovanadate enhanced overall phosphorylation, but no difference was apparent between untransfected and HMTK1-transfected cells (Figure S4B). To examine whether any pTyr-containing proteins in SYF cells bound to HMTK1, we isolated HMTK1 and associated proteins using FLAG beads and analyzed them by anti-pTyr Western blotting. We did not observe any significant pTyr-containing bands in these experiments (Figure S5). Expression of HMTK1 in COS-7 cells gave similar results (data not shown). Because there was no evidence for HMTK1 autophosphorylation, HMTK1 may not be phosphorylated in the activation loop to significant levels. Thus, although HMTK1 possesses intrinsic tyrosine kinase activity (Figs. 1 and 2), its activity in mammalian cells is undetectable by standard anti-phosphotyrosine Western blotting.

**Figure 4:**
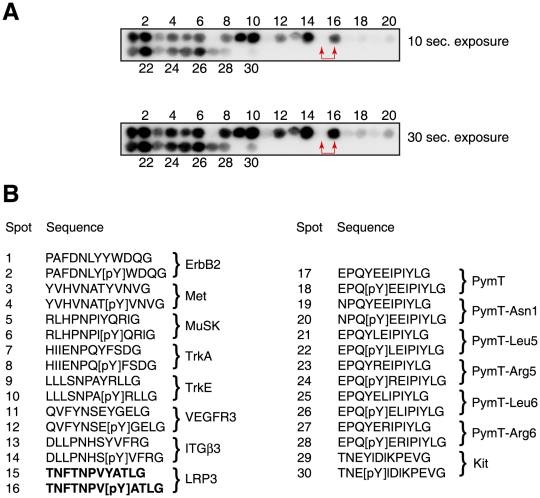
Peptide array experiment. (A) A PepSpots array containing 30 immobilized peptides was probed with purified biotinylated HMTK1 PTB domain. Bound PTB domain was detected using streptavidin-HRP and enhanced chemiluminescence. Exposures from 10 and 30 seconds are shown for dynamic range comparisons. Peptides 15 and 16, selected for further study, are indicated with red arrowheads. (B) Sequences of the immobilized tyrosine- and phosphotyrosine-containing peptides. The proteins from which the peptides are derived are listed to the right of the sequences. Peptides 17–28 contain the polyoma middle T (PymT) sequence (wild-type and single amino acid variants). Peptides 15–16 from LRP3, chosen for further study, are shown in bold.

Different metazoan PTB domains have different ligand preferences [26], and it is not currently possible to predict PTB specificity from amino acid sequence alone [15]. The third PTB domain of HMTK1 bound to the typical SH2 ligand pYEEI (Fig. 3), although this sequence does not conform to the typical PTB ligand. To search more broadly for HMTK1 PTB ligands, we carried out experiments using a peptide array with multiple potential PTB ligands. The PTB domain was biotinylated by co-expression with biotin ligase in *E. coli* [19]. Purified biotinylated PTB domain was then used to probe a membrane on which 30 potential binding peptides had been immobilized (Fig. 4A). The peptide sequences in Fig. 4B include phosphorylated and unphosphorylated counterparts known as targets for different classes of mammalian PTB domains [26]. This experiment identified several HMTK1 PTB3 binding sequences. Some peptides (e.g., spots 1–2) bound with similar affinity in their phosphorylated and unphosphorylated states. Other peptide pairs (e.g., spots 7–8, 11–12, 15–16) showed stronger binding when tyrosine phosphorylated (Fig. 4A).

**Figure 5:**
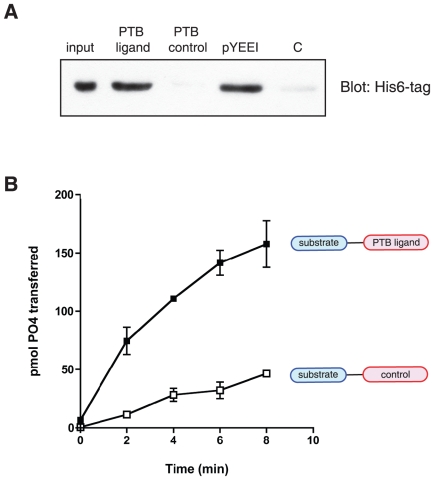
Substrate targeting by PTB ligand. (A) Purified HMTK1 (6.6 µg) was incubated with PTB ligand-containing substrate (KKAEEEIYGEFEANFTNPVpYATLG), PTB control substrate (KKAEEEIYGEFEANFTNPVFATLG), pYEEI peptide, or control resin in final volumes of 60 µl. After washing, bound proteins were eluted with SDS-PAGE sample buffer and analyzed by Western blotting with anti-His_6_-tag antibody. (B) The kinase activity of HMTK1 (300 nM) toward the PTB ligand-containing substrate or PTB control substrate (shown schematically) was determined using the phosphocellulose paper assay. The reaction contained 20 mM Tris-HCl (pH 7.4), 10 mM MgCl_2_, 0.2 mM ATP, 30 µM peptide, and [γ-^32^P]-ATP. At each time point, an aliquot was withdrawn and 10% trichloroacetic acid was added to stop the reaction. Incorporation of ^32^P into the peptides was determined by scintillation counting.

We selected a pair of peptides for further study. The peptides (spots 15–16, containing the sequence TNFTNPVYATG, derived from the low density lipoprotein-3 receptor), showed binding to the biotinylated PTB domain that was strongly phosphotyrosine-dependent (Fig. 4A). We synthesized an individual peptide in which the sequence NFTNPVpYATG was connected to a tyrosine kinase substrate sequence. As a control, we prepared a peptide in which the pTyr residue was replaced with Phe (we did not use Tyr in the control sequence to avoid complications due to a second phosphorylatable tyrosine in the substrate). We immobilized the two peptides on Affi-Gel resin, and tested binding to the purified HMTK1 protein. HMTK1 bound to the pTyr-containing sequence, but binding to the Phe-containing control peptide was undetectable (Fig. 5A). Next, we carried out substrate targeting experiments similar to those shown above in Fig. 2B and C. HMTK1 preferentially phosphorylated the pTyr-containing peptide as compared to the control (Fig. 5B). Steady-state kinetic analyses of these peptides gave a K_m_ value of 33 µM for the pTyr peptide and 450 µM for the Phe peptide. The value of V_max_ for the pTyr-peptide (4.9 µmol/min/mg enzyme) is comparable to the value for Src family kinases with the same peptide (e.g., Hck, with V_max_ = 3.0 µmol/min/mg enzyme [24]). The difference in K_m_ values between pTyr- and Phe-containing peptides is comparable to the well-studied effect of the SH2 domain in Src kinase substrate recognition [24], [27], suggesting that the PTB domain of *Monosiga brevicollis* HMTK1 may function as a substrate targeting module.

## Discussion

Metazoan nonreceptor tyrosine kinases invariably possess noncatalytic modular domains in addition to their conserved catalytic domains. These noncatalytic regions play important roles in subcellular localization, enzymatic regulation, protein-protein interactions, and substrate recognition [8]. The kinome of the choanoflagellate *Monosiga brevicollis* likewise shows numerous examples of tyrosine kinase catalytic domains connected to modular signaling domains [2], [3]. Strikingly, many of the domain combinations found in *Monosiga* have not been seen elsewhere, including in metazoans. The noncatalytic regions of these kinases presumably have important functional roles, similar to their metazoan counterparts.

Here, we have carried out a biochemical study of one such kinase, HMTK1. Ten of the fifteen *Monosiga* HMTK kinases contain at least one PTB domain, although some of the domains are small and may not fold into a functional unit. Although the number of PTB domains varies in this family (1–3 domains), it is noteworthy that the PTB domains all lie N-terminal to the kinase domains, i.e., in the same relative position as SH2 domains and kinase domains in Src-family kinases. In Src, the positioning of the SH2 domain relative to the kinase domain plays a role in substrate recognition [28]. A mutant form of Src in which the SH2 domain was placed C-terminal to the catalytic domain showed decreased phosphorylation of Cas and Sam68 in cells, and decreased phosphorylation of pYEEI-containing peptide substrates *in vitro* [28].

The combination of a PTB and a tyrosine kinase domain is not found in higher metazoans. On the other hand, the individual PTB and kinase domains of HMTK1 show sequence conservation with related domains from metazoans. The third PTB domain of HMTK1 (as well as the first and second PTB domains, not studied here) are related to the Numb and Gulp families of PTB domains (Fig. S3). PTB domains from other *Monosiga* HMTK kinases (e.g., HMTK4, HMTK8) show the most similarity to these same families of PTB domains, suggesting that HMTK1 is fairly typical of the family in this regard. The HMTK1 catalytic domain is most closely related to mammalian receptor tyrosine kinase domains.

We confirmed that HMTK1 possesses tyrosine kinase activity by expressing the protein in insect cells, purifying it, and measuring phosphorylation of synthetic peptides. To examine HMTK1 activity in intact cells, we turned to a heterologous cell system (mammalian SYF fibroblasts), due to the difficulties associated with expressing genes in *Monosiga*. The activity of HMTK1 was undetectable in these cells (Fig. S3). We also explicitly tested the mammalian receptor tyrosine kinases ErbB2 and IGF1R as potential binding partners, but HMTK1 failed to bind or phosphorylate these proteins when co-expressed with them (data not shown). One possible explanation is that the substrate specificity of HMTK1 is tuned to proteins in *Monosiga* cells, rather than mammalian proteins. Alternatively, the protein may adopt a low-activity conformation in mammalian cells through autoinhibitory interactions, or though interactions with other cellular proteins. (For example, HMTK1 may normally require activation by other *Monosiga* kinases which are absent in mammalian cells, and the high activity of mammalian tyrosine phosphatases would repress HMTK1 activity). Our data cannot distinguish between these possibilities at present.

We carried out experiments with immobilized peptide arrays to search more broadly for HMTK1 binding partners. Pawson and coworkers previously used NPXY peptide arrays to screen for binding partners for 10 diverse PTB domains [26]. The results showed a variety of pTyr-dependent and pTyr-independent interactions with the various classes of PTB domains. For our experiments, we selected representative peptide sequences that bound to the different classes of PTB domains. We included peptide sequences with phosphorylated and unphosphorylated tyrosine. Because our preliminary data showed binding of the HMTK1 PTB domain to the sequence pYEEI, which is found in the polyoma virus middle T antigen, we also included the wild-type middle T sequence and several variants within the YEEI motif. The HMTK1 PTB domain bound to many of the peptides in the array (Fig. 4). Several of the NPXY-type sequences bound more strongly to the HMTK1 PTB than the pYEEI sequence. Some of the sequences did not show significant pTyr-dependence, probably due to a high background from hydrophobic interactions. We examined one sequence (TNFTNPVYATLG, derived from the LRP3 receptor) which bound much more strongly in the phosphorylated state. We showed that a synthetic peptide containing this sequence (with pTyr) bound to the longer PTB-kinase construct of HMTK1 (Fig. 5A). Furthermore, we demonstrated that this peptide was phosphorylated more efficiently than a control in which pTyr was replaced by Phe (Fig. 5B). The presence of pTyr led to a ≈15-fold reduction in peptide K_m_, which is comparable to the effects of the pYEEI motif recognized by the Src SH2 domain.

These results suggest that the role of the HMTK1 PTB domain may be to target the enzyme to potential substrates in *Monosiga brevicollis* cells. To identify potential substrates, we searched for occurrences of the TNFTNPVYATLG motif within the *Monosiga* genome using the protein-translated nucleotide BLAST search tool on the genome site (http://genome.jgi-psf.org/Monbr1/Monbr1.home.html). Although there were no matches to the full sequence, two predicted proteins had partial matches. Gene model number 11339 encodes a predicted transmembrane protein with cadherin and SH2 domains; the predicted protein includes a FSNPMYA sequence. A second SH2-containing *Monosiga* protein (gene model number 34447) contains the sequence NPVYA. We carried out a similar analysis using other PTB-binding peptides from the array experiment. The sequence EYGEL from *Monosiga* RTKB8 was identified by a search using peptides 11/12 (Fig. 4A), with the sequence QVFYNSEpYGEL. It will be interesting to determine whether these proteins are phosphorylated in *Monosiga* cells.

Many metazoan Ser/Thr and Tyr protein kinases recruit their substrates via interactions with secondary binding sites (i.e., apart from the kinase catalytic domain). MAP kinases, for example, possess distal docking sites that enhance substrate specificity [29]. In nonreceptor tyrosine kinases, several modular protein-protein interaction domains have been found to influence substrate specificity, including SH2, SH3, PH, F-actin binding, and focal adhesion binding domains. The results reported here for the primitive kinase HMTK1 expand the list to include PTB domains, and together with previous results for MbSrc1 [13] and MbSrc4 [14], suggest that the domain architectures in tyrosine kinases evolved first to fill this substrate recruitment function. Further evolutionary fine-tuning of the domain arrangements was necessary to establish the elaborate mechanisms of regulation needed in metazoan signaling pathways.

## Supporting Information

Figure S1 **Sequence alignment of the kinase domains of HMTK1 and human fibroblast growth factor receptor 1.** The HRD/HMD sequences in the catalytic loop are underlined, and the tyrosine residue in the predicted activation loop is shown in red.
(EPS)

Figure S2 **Autophosphorylation of HMTK1.** Purified HMTK1 (36 ng) was analyzed by anti-pTyr Western blotting after incubation with Yersinia tyrosine phosphatase (+YOP), with no treatment (HMTK), or after an autophosphorylation reaction (+ATP). The autophosphorylation reaction contained 1 mM ATP and 10 mM MgCl_2_, and proceeded for 45 minutes at 30°C.
(EPS)

Figure S3 **Structural model of HMTK1 PTB.** A homology model of the third PTB domain of HMTK is shown, based on the human Numb-R protein [30]. We identified Numb-R as the closest relative to the third PTB domain of HMTK using HMM-HMM comparisons (http://toolkit.tuebingen.mpg.de/hhpred). Numb-R contains two Phe residues that are important for ligand binding [31], and the two Phe residues are conserved at equivalent positions (F71 and F117) in the HMTK PTB domain.
(EPS)

Figure S4 **Expression of HMTK1 in mammalian cells.** (A). SYF cells were transfected with FLAG-tagged HMTK1 (wild-type, ΔPTB, or mutant forms, as indicated). Whole cell lysates were analyzed by SDS-PAGE and Western blotting with anti-pTyr, anti-FLAG, or anti-tubulin antibodies, as indicated. C = untransfected SYF cells. The figure is representative of 4 experiments. (B) Similar experiments were carried out on control or HMTK1-transfected SYF cells treated with 0. 0.1, or 1.0 mM sodium orthovanadate (20 minutes, 37°C) prior to lysis. Similar amounts of lysates were used in panels (A) and (B), but a shorter exposure time was used in (B) than in panel (A).
(EPS)

Figure S5 **Immunoprecipitation experiment.** HMTK1 (wild-type or mutants) was immunoprecipitated from SYF cells using anti-FLAG affinity resin. Proteins in the immunocomplexes were separated by SDS-PAGE and analyzed by Western blotting with anti-pTyr and anti-FLAG antibodies. The band at ≈110 kDa is a non-specific band. The figure is representative of 3 experiments.
(EPS)
